# Supplementary material for: Proton-controlled molecular ionic ferroelectrics
Source: Nat Commun. 2023 Aug 19;14:5041. doi: 10.1038/s41467-023-40825-6 (PMC10439891; doi:10.1038/s41467-023-40825-6)
Supplement: Supplementary file 1 — Supplementary information [file 41467_2023_40825_MOESM1_ESM.pdf]

Supplementary Information

Proton-controlled Molecular Ionic Ferroelectrics

Yulong Huang[1,*], Jennifer L. Gottfried[2], Arpita Sarkar[1], Gengyi Zhang[3], Haiqing Lin[3], Shenqiang Ren[1,4,5,6,*]

[1]*Department of Mechanical and Aerospace Engineering, University at Buffalo, The State University of New York, Buffalo, NY, 14260, USA*
[2]*Weapons Sciences, US Army Combat Capabilities Development Command-Army Research Laboratory, Aberdeen Proving Ground, Aberdeen, MD 21005, USA*
[3]*Department of Chemical and Biological Engineering, University at Buffalo, The State University of New York, Buffalo, NY, 14260, USA*
[4]*Department of Chemistry, University at Buffalo, The State University of New York, Buffalo, NY, 14260, USA*
[5]*Research and Education in Energy, Environment and Water (RENEW) Institute, University at Buffalo, The State University of New York, Buffalo, NY, 14260, USA*
[6]*Department of Materials Science and Engineering, University of Maryland, College Park, MD 20742, USA*

*E-mail: yhuang59@buffalo.edu; shenren@buffalo.edu

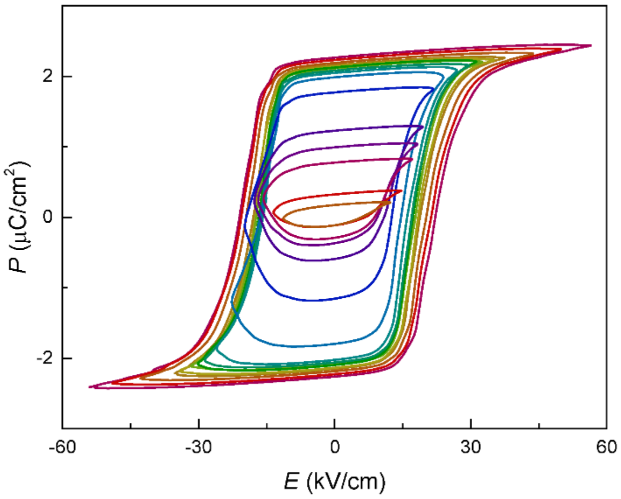

Figure S1. Polarization-electric field (P-E) hysteresis loops of $ImClO_4$ crystal. The *P*-*E* loops expand with the increasing electric field bias, then gradually saturate at a large electric field.

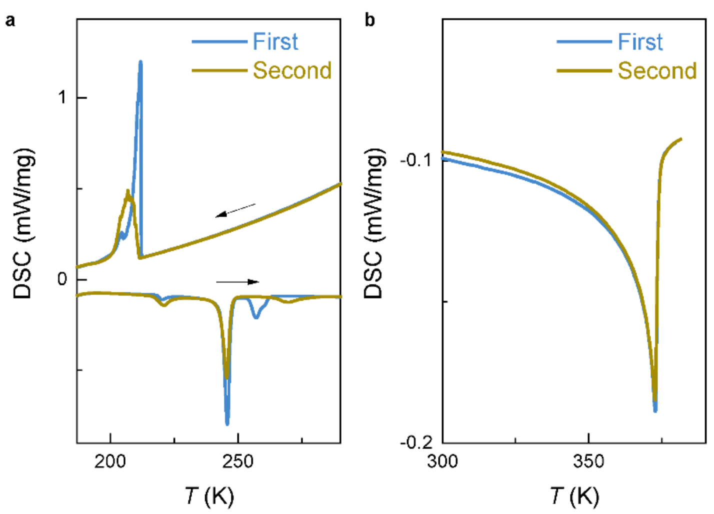

Figure S2. Differential scanning calorimetry (DSC) scans of $ImClO_4$. (a) Two low-temperature exothermic (212 K) and endothermic (246 K) peaks indicate the structural transition during cooling and heating. The shape of DSC peaks measured in the second time become wider and weaker compared to those measured in the first time. (b) The endothermic peak at 373 K keeps constant during repeated measurements, corresponding to ferroelectric transition.

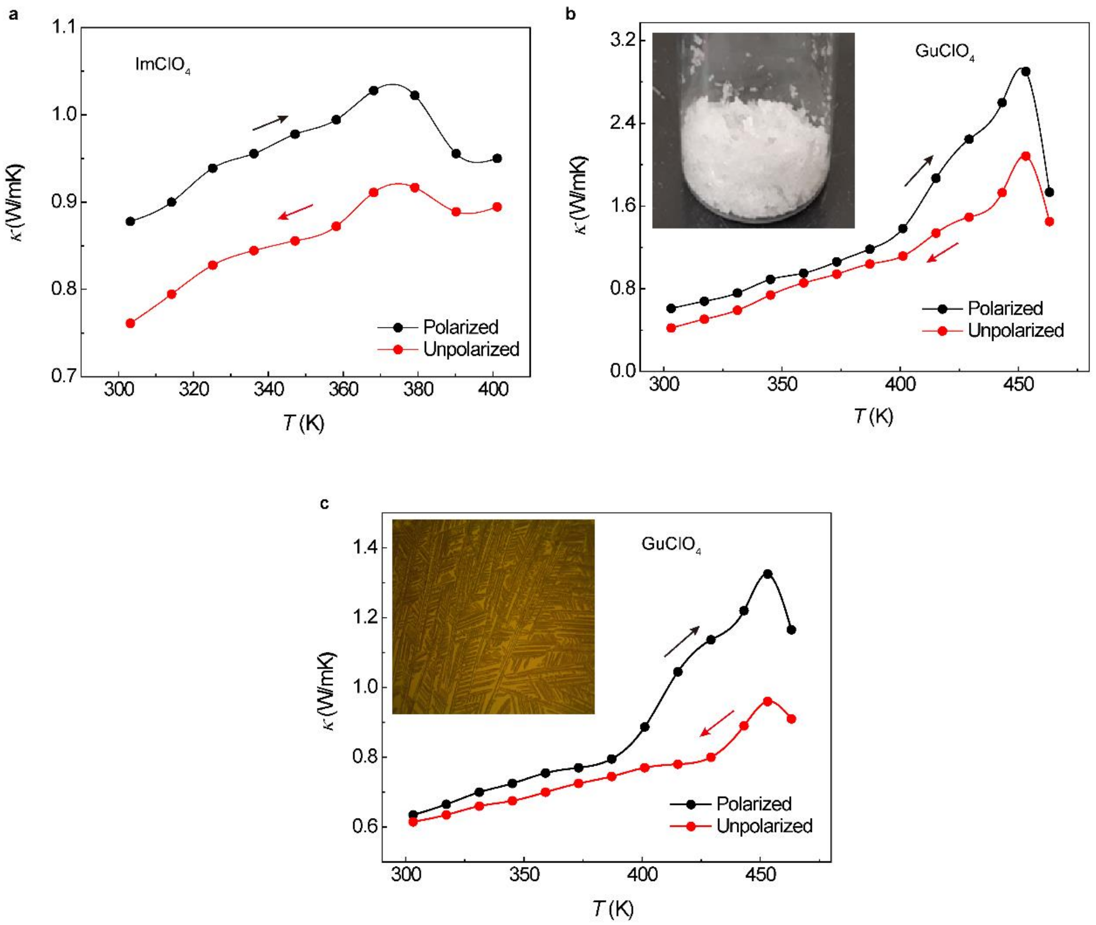

Figure S3. Polarization effect on temperature-dependent thermal conductivity via heating (black) and cooling (red) processes. (a) A maximum of thermal conductivity of $ImClO_4$ powder appears at around 370 K (near Curie temperature) in both initial polarized state (heating from 300 K) and initial unpolarized state (cooling from 400 K). The thermal conductivity of an initial polarized state is higher than that of an initial unpolarized state. (b) The thermal conductivity of guanidinium perchlorate ($GuClO_4$) powder follows a polarization effect similar to $ImClO_4$, while the maximum occurs at around 450 K. The difference of thermal conductivity measured by heating and cooling is enhanced at above 400 K. (c) The film sample of $GuClO_4$ presents the same result with powder sample, regarding the polarization effect on thermal conductivity.

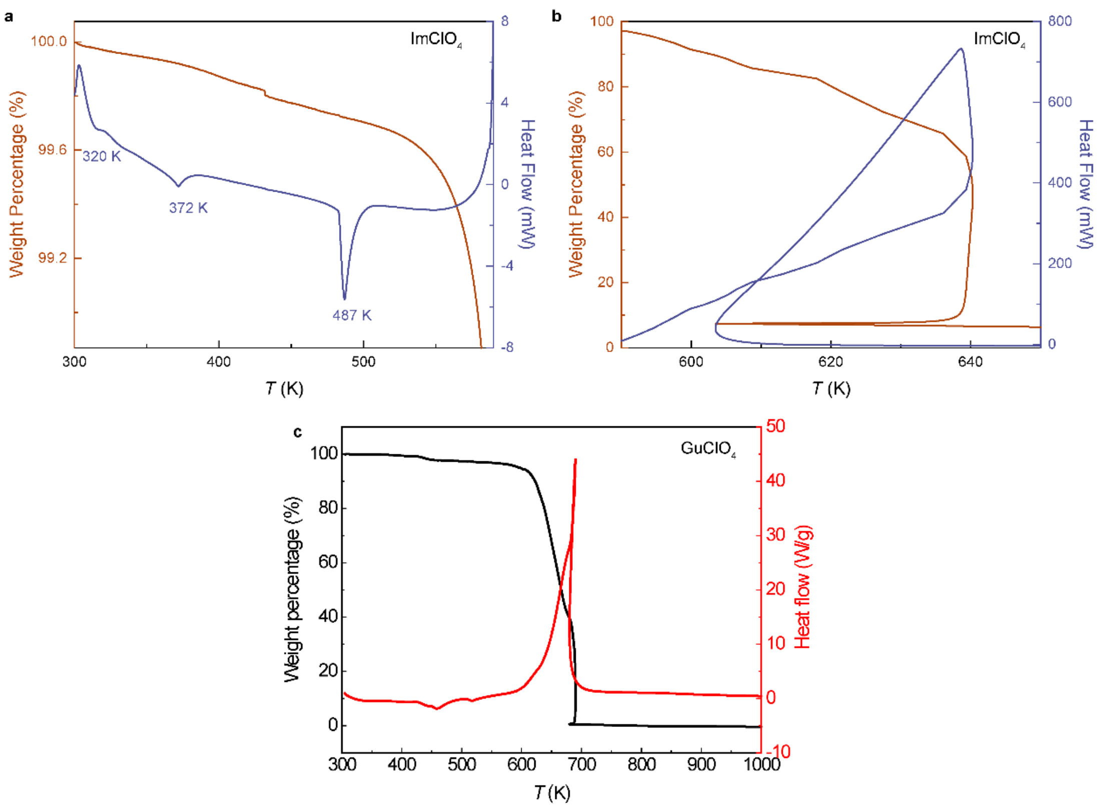

Figure S4. Thermogravimetric analysis (TGA) of molecular ionic ferroelectrics. (a) Two obvious endothermic peaks in $ImClO_4$ occur at 372 K and 487 K. (b) A large weight loss in $ImClO_4$ happens at around 640 K with an exothermic anomaly. (c) $GuClO_4$ presents a small endothermic peak at around 450 K and starts to decompose at around 600 K.

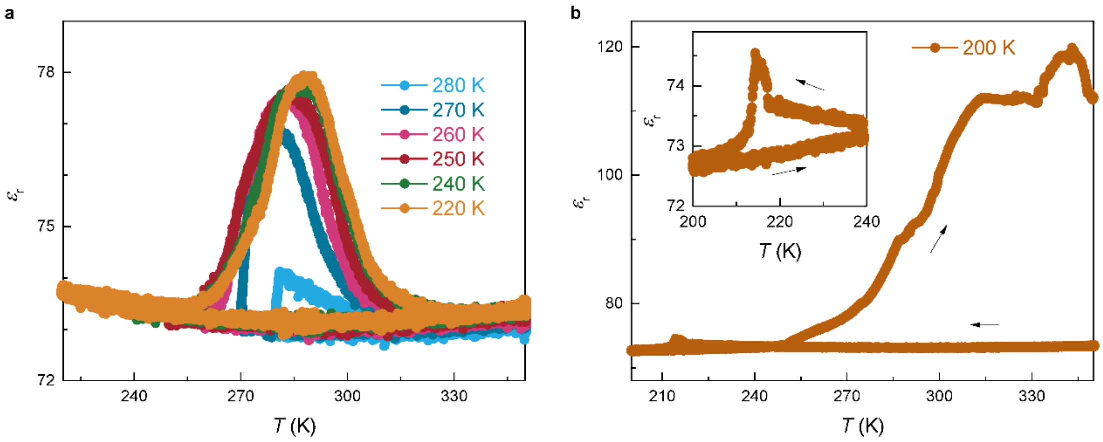

Figure S5. Temperature-dependent relative permittivity measured by cooling from 350 K to *T* and heating from T to 350 K at 10 kHz. (a) The permittivity peak occurs at the heating process and depends on the lowest measured temperature *T*. When T varies from 280 K to 220 K, the permittivity peak is enhanced and shifts to higher temperature. (b) The relative permittivity measured from 350 K to 200 K by cooling and heating. The permittivity anomaly is largely enhanced.

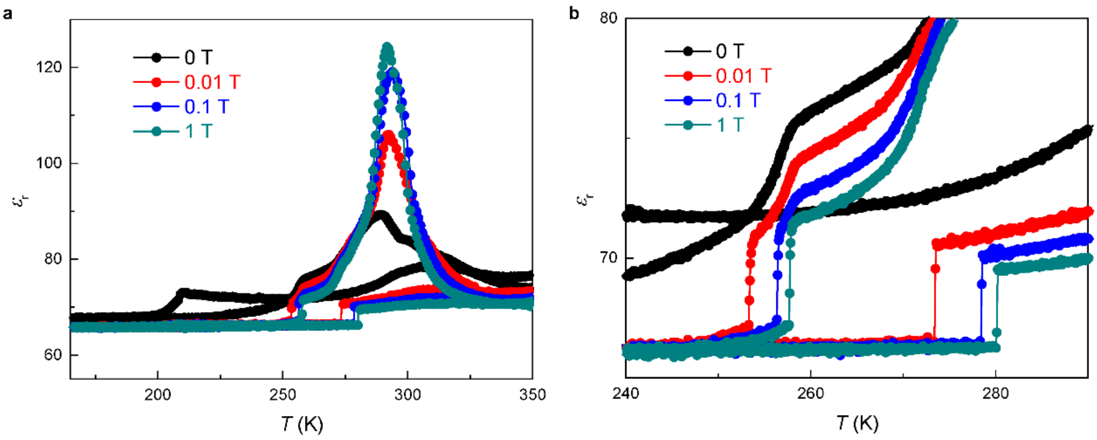

Figure S6. Magnetic field effect on relative permittivity of ImClO$_4$ crystal. (a) The relative permittivity is measured from 350 K to 165 K by cooling and heating. Magnetic field is applied with 0 T, 0.01 T, 0.1 T, and 1 T. The permittivity anomaly is enhanced by magnetic field. (b) A sharp jump in permittivity occurs at 273 K when magnetic field is 0.01 T, and shifts to higher temperature 279 K (0.1 T) and 280 K (1 T).

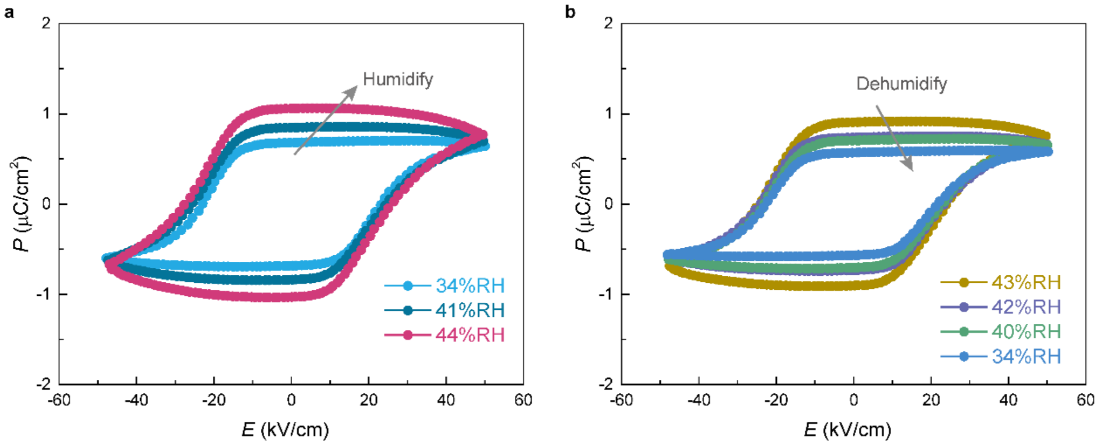

Figure S7. Humidity effect on polarization-electric field (P-E) hysteresis loops of $ImClO_4$ crystal. (a) P-E loops expand with increasing relative humidity from 34% to 44 %. (b) P-E loops gradually recover into initial shape by dehumidifying the environment of $ImClO_4$ crystal.

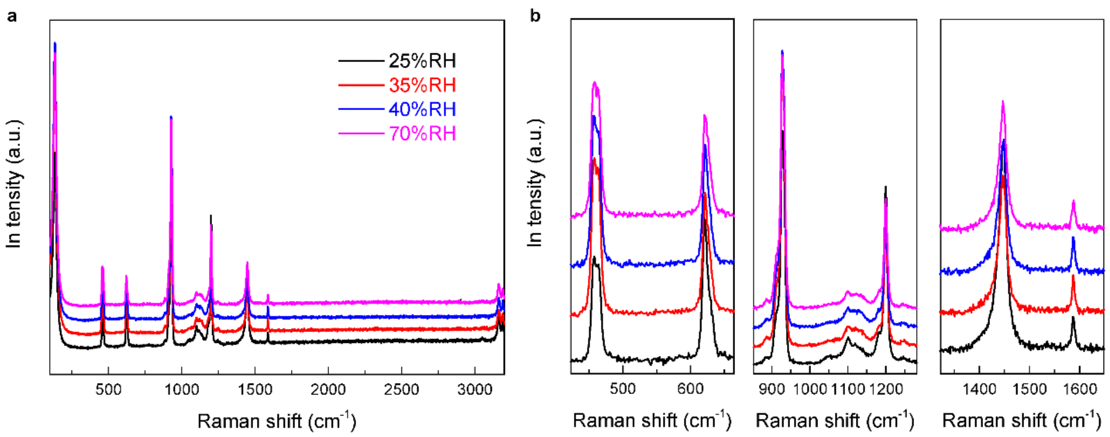

Figure S8. Raman spectra of $ImClO_4$ crystal under different relative humidity. (a) Raman spectra were measured from 3200 cm^{-1} to 100 cm^{-1}. The relative humidity varies from 25 %, 35 %, 40 %, to 70 %. (b) Enlarged Raman spectra at different Raman shift ranges indicate no obvious changes by humidity.

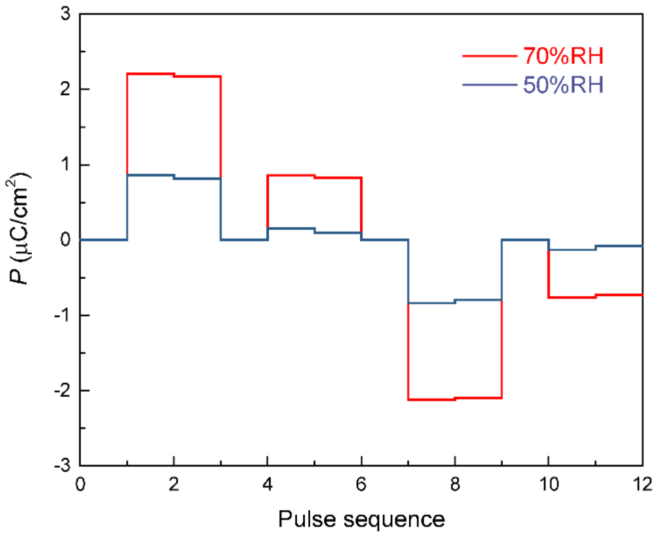

Figure S9. PUND measurement on $ImClO_4$ crystal at different humidity.

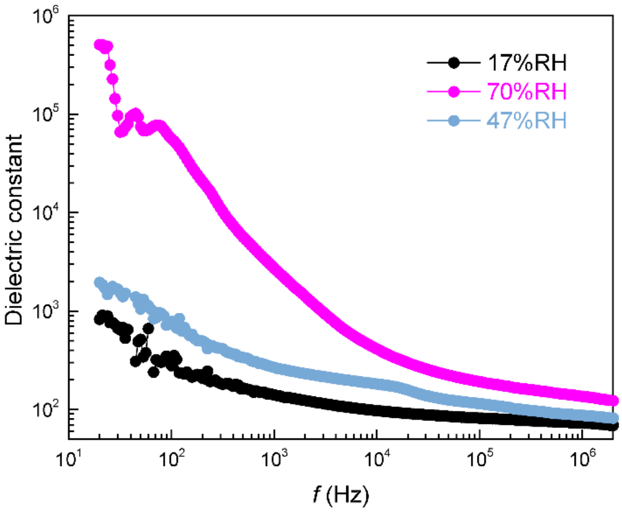

Figure S10. Frequency dependent dielectric constant under different humidity.

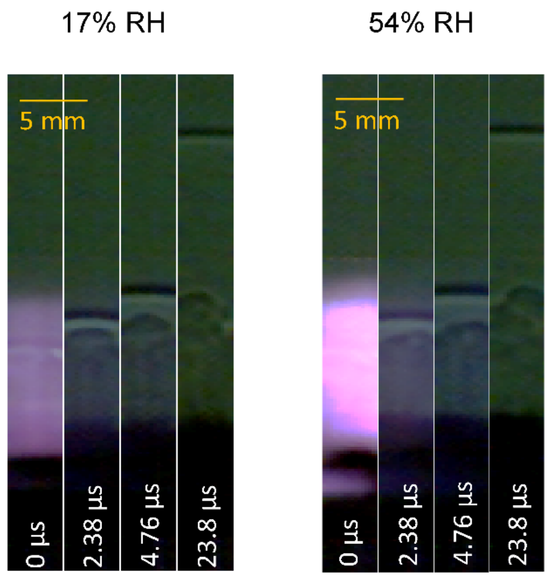

Figure S11. Selected high-speed schlieren video snapshots from laser-shocked $ImClO_4$ crystal in different humidity.

Table S1. Laser-induced shock velocity and estimated detonation velocity of $ImClO_4$.

$ImClO_4$	Laser-induced shock velocity (m/s)	Estimated detonation velocity (km/s)
Polarized [1]	740.01 ± 11.14	7.20 ± 0.27
Depolarized [1]	738.33 ± 8.37	7.16 ± 0.21
17%RH	729.13 ± 7.82	7.21 ± 0.22
54%RH	731.00 ± 8.21	7.26 ± 0.24

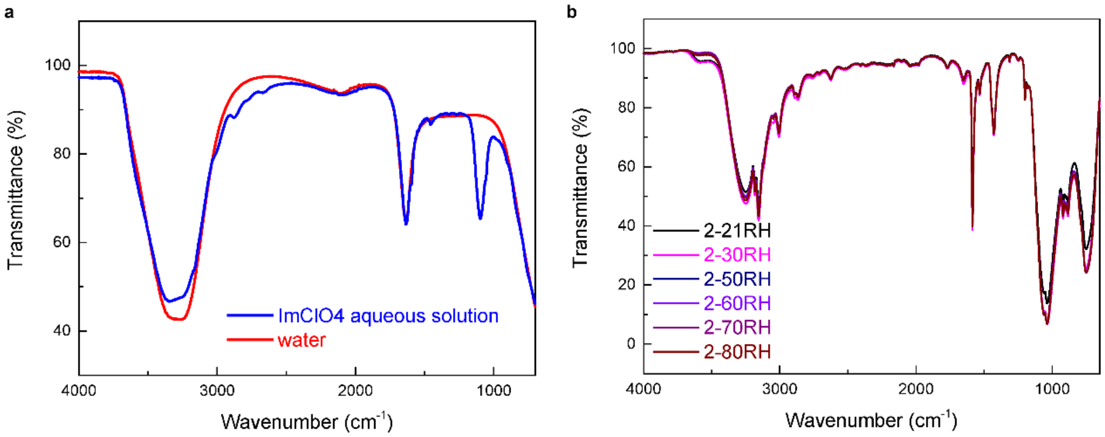

Figure S12. Fourier-transform infrared spectra of $ImClO_4$. (a) The FTIR spectrum of $ImClO_4$ aqueous solution shows an additional peak at 1094 cm^{-1} compared to water. (b) The FTIR spectra of $ImClO_4$ crystal indicate a lower transmittance by increasing humidity.

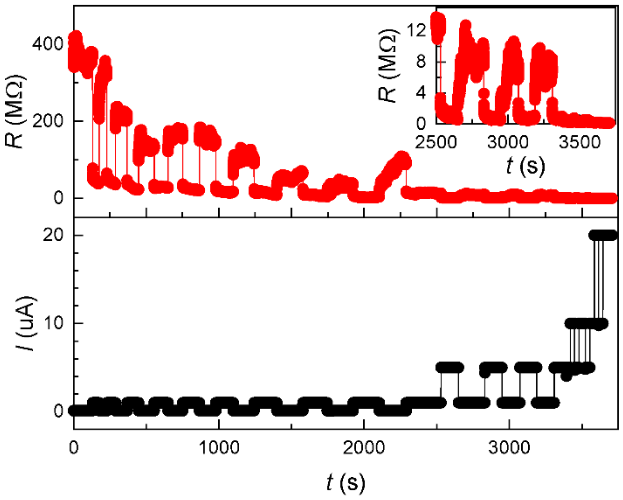

Figure S13. Time-dependent current switching tunes resistance of $ImClO_4$ crystal. Resistance keeps decreasing when applied current is switched among 0.1 uA, 1 uA, 5 uA, 10 uA, and 20 uA.

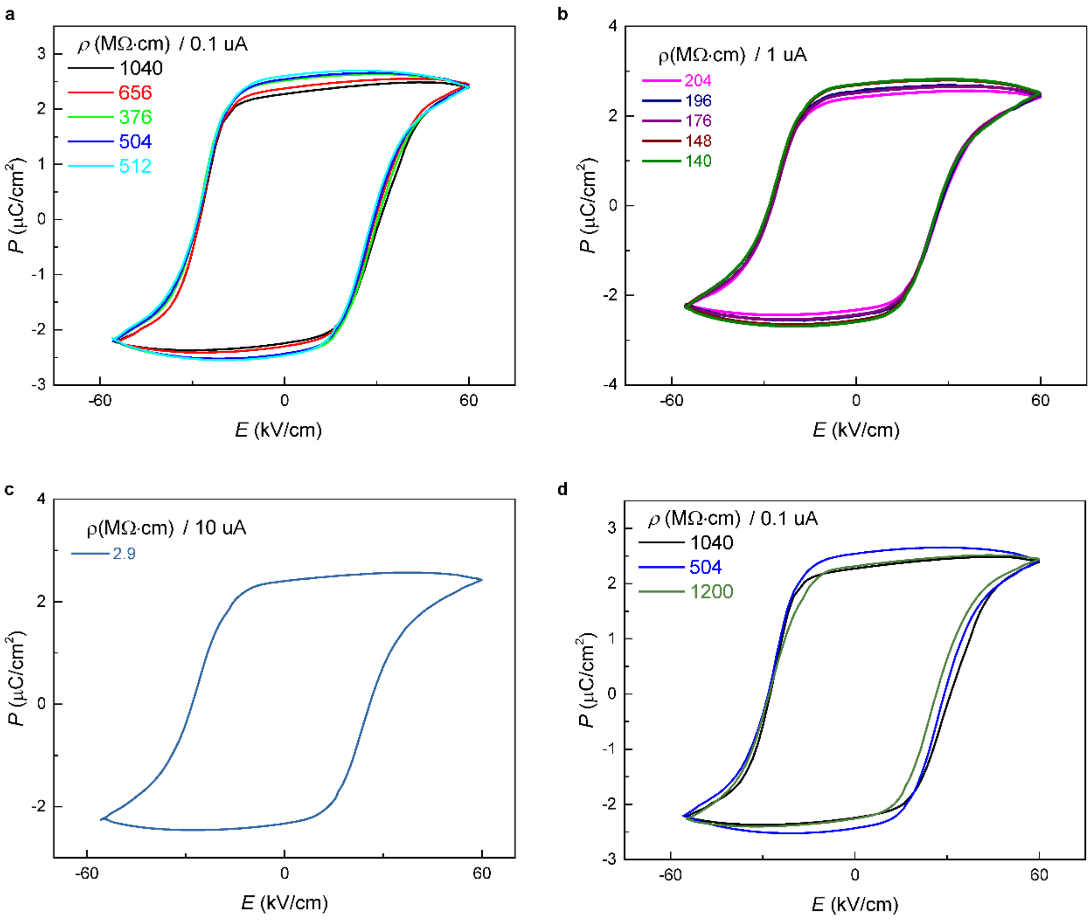

Figure S14. Polarization-electric field (P-E) hysteresis loops of $ImClO_4$ crystal measured at different resistive states. (a) P-E loops are enlarged by decreasing the resistivity of $ImClO_4$ when a constant current of 0.1 uA is applied. (b) With continuous decrease of resistivity by applying 1 uA current, P-E loops keep to expand. (c) The applicable current is up to 10 uA, while $ImClO_4$ crystal still show high ferroelectric performance in a large reduced resistivity. (d) ImClO4 crystal recovered into a high resistivity (1200 MΩ·cm) when a low current was applied again, meanwhile corresponding P-E loop shrunk back to the initial shape.

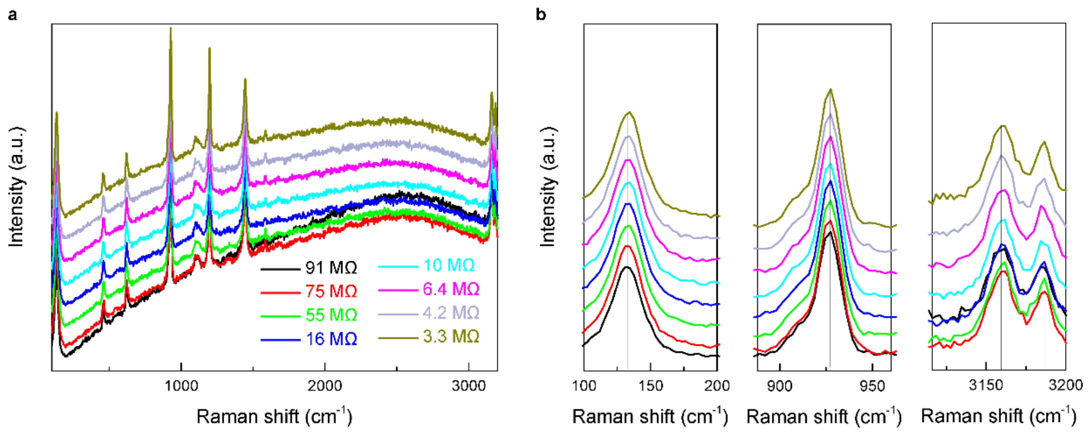

Figure S15. Raman spectra of $ImClO_4$ crystal at different resistive states from 91 MΩ to 3.3 MΩ. (a) The whole Raman spectra profiles are not varied by the resistive state. (b) The enlarged Raman peaks are not shifted by the resistive states.

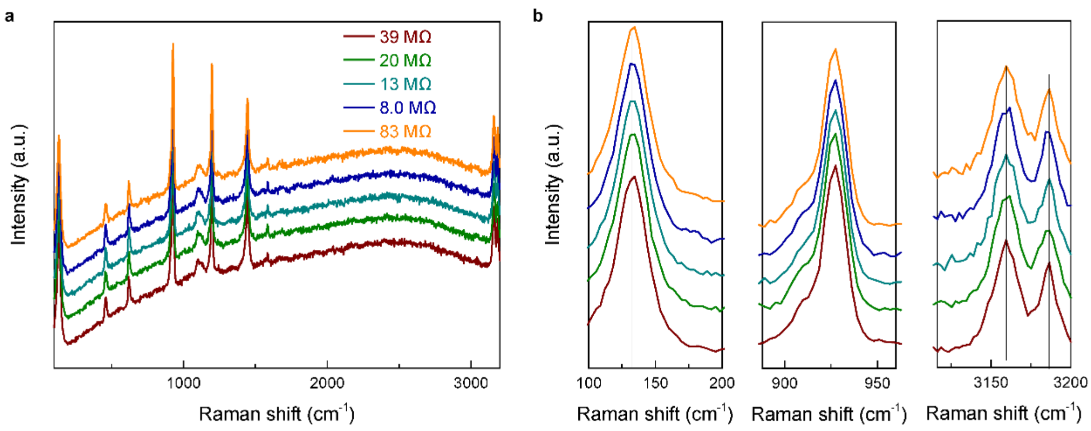

Figure S16. Raman spectra of $ImClO_4$ crystal at different resistive states from 39 MΩ to 7.0 MΩ, and back to initial 83 MΩ. (a) The whole Raman spectra profiles are not varied by the resistive state. (b) The enlarged Raman peaks are not shifted by the resistive states.

Reference

1. Hu, Y. *et al.* Releasing chemical energy in spatiallyprogrammed ferroelectrics. *Nat. Commun.* **13**, 6959 (2022).
